# Magnetic resonance imaging pre and post pulmonary vein isolation for atrial fibrillation: diagnostic accuracy to detect and characterize ablation lesions

**DOI:** 10.1186/1532-429X-14-S1-P207

**Published:** 2012-02-01

**Authors:** Daniel A Jones, Ross J Hunter, Redha Boubertakh, Louisa Malcolme-Lawes, Prapa Kanagaratnam, Nicholas S Peters, Christoph Juli, Victoria Baker, Mark Earley, Simon Sporton, Ceri Davies, Mark Westwood, Richard J Schilling, Steffen E Petersen

**Affiliations:** 1 NIHR Cardiovascular Biomedical Research Unit, Barts and the London NHS Trust, London, UK; 2 International Centre for Circulatory Health, St Marys Hospital, Imperial College Healthcare NHS Trust., London, UK

## Summary

LGE imaging of left atrial scar is promising and can detect pre/post ablation procedures however further quality control required to accurately depict lesion distribution

## Background

We tested the hypothesis that cardiovascular magnetic resonance (CMR) imaging can reliably distinguish the presence or absence of ablation lesions by blinded analysis of pre and post ablation imaging.

## Methods

Consecutive patients with paroxysmal AF in a randomised study comparing pulmonary vein isolation by wide area circumferential radiofrequency ablation (WACA) to ostial ablation with a cryo-balloon (CRYO) underwent CMR late gadolinium enhancement (LGE) imaging pre- and 3 months post ablation. Imaging was anonymized for blinded analysis of (1) LGE images, and (2) a 3D fusion image with LGE projected onto a segmented LA surface (thresholding set at 5 SD above mean ventricular signal). Scans were categorised using both assessment techniques separately as pre or post ablation, and if post ablation, whether lesions were in an ostial or WACA distribution.

## Results

LGE imaging was performed in 50 patients (aged 61 ± 10 years, 72% male). Sensitivity and specificity for detection of ablation lesions was 60% and 96% on LGE imaging, or 88% and 82% respectively on 3D fusion imaging. Detection of WACA and CRYO lesion sets were correct in 14/24 and 16/26 respectively on LGE imaging, or 21/24 and 23/26 on 3D fusion imaging. Assessment of lesion distribution was correct for 13/50 from LGE images (4/24 for WACA and 9/26 for CRYO) and 26/50 from 3D fusion images (p = 0.013; 13/24 for WACA and 13/26 for CRYO).

## Conclusions

LGE imaging of atrial scar after ablation therapy is feasible. The technique still needs quality control standards established in order to determine the appropriate SD setting and to judge whether lack of peri-ostial scar is due to a sub-optimal scan or myocardial recovery

## Funding

National Institute of Health Research (UK).

**Table 1:** Diagnostic accuracy of detecting atrial scarring in blinded analysis of pre and post ablation MR images.

	Sensitivity (%)	Specificity (%)	PPV (%)	NPV (%)
Late Gadolinium Enhancement	60	96	94	68
Segmented Fusion images	88	82	81	87

**Table 2:** Accuracy of MR images to locate atrial scarring according to ablation technique

Correct identification of lesion distribution	LGE	Fusion segmented image
WACA	4/24 (17%)	13/24 (54%)
Cryo	9/26 (35%)	13/26 (50%)

